# Severe anaemia, iron deficiency, and susceptibility to invasive bacterial infections

**DOI:** 10.12688/wellcomeopenres.18829.1

**Published:** 2023-02-02

**Authors:** Kelvin M. Abuga, Manfred Nairz, Calman A. MacLennan, Sarah H. Atkinson

**Affiliations:** 1 Kenya Medical Research Institute (KEMRI) Centre for Geographical Medicine Research-Coast, KEMRI-Wellcome Trust Research Programme, Kilifi, 80108, Kenya; 2 Open University, KEMRI-Wellcome Trust Research Programme – Accredited Research Centre, Kilifi, 80108, Kenya; 3 Department of Internal Medicine II, Medical University of Innsbruck, Innsbruck, 6020, Austria; 4 Jenner Institute, Nuffield Department of Medicine, University of Oxford, Oxford, OX3 7DQ, UK; 5 Centre for Tropical Medicine and Global Health, Nuffield Department of Medicine, University of Oxford, Oxford, OX3 7LG, UK; 6 Department of Paediatrics, University of Oxford, Oxford, OX3 9DU, UK

**Keywords:** severe anaemia, hepcidin, iron deficiency, vaccines, nutritional immunity, macrophages, neutrophils, bacteraemia, Salmonella

## Abstract

Severe anaemia and invasive bacterial infections remain important causes of hospitalization and death among young African children. The emergence and spread of antimicrobial resistance demand better understanding of bacteraemia risk factors to inform prevention strategies. Epidemiological studies have reported an association between severe anaemia and bacteraemia. In this review, we explore evidence that severe anaemia is associated with increased risk of invasive bacterial infections in young children. We describe mechanisms of iron dysregulation in severe anaemia that might contribute to increased risk and pathogenesis of invasive bacteria, recent advances in knowledge of how iron deficiency and severe anaemia impair immune responses to bacterial infections and vaccines, and the gaps in our understanding of mechanisms underlying severe anaemia, iron deficiency, and the risk of invasive bacterial infections.

## Introduction

**Figure 1.**
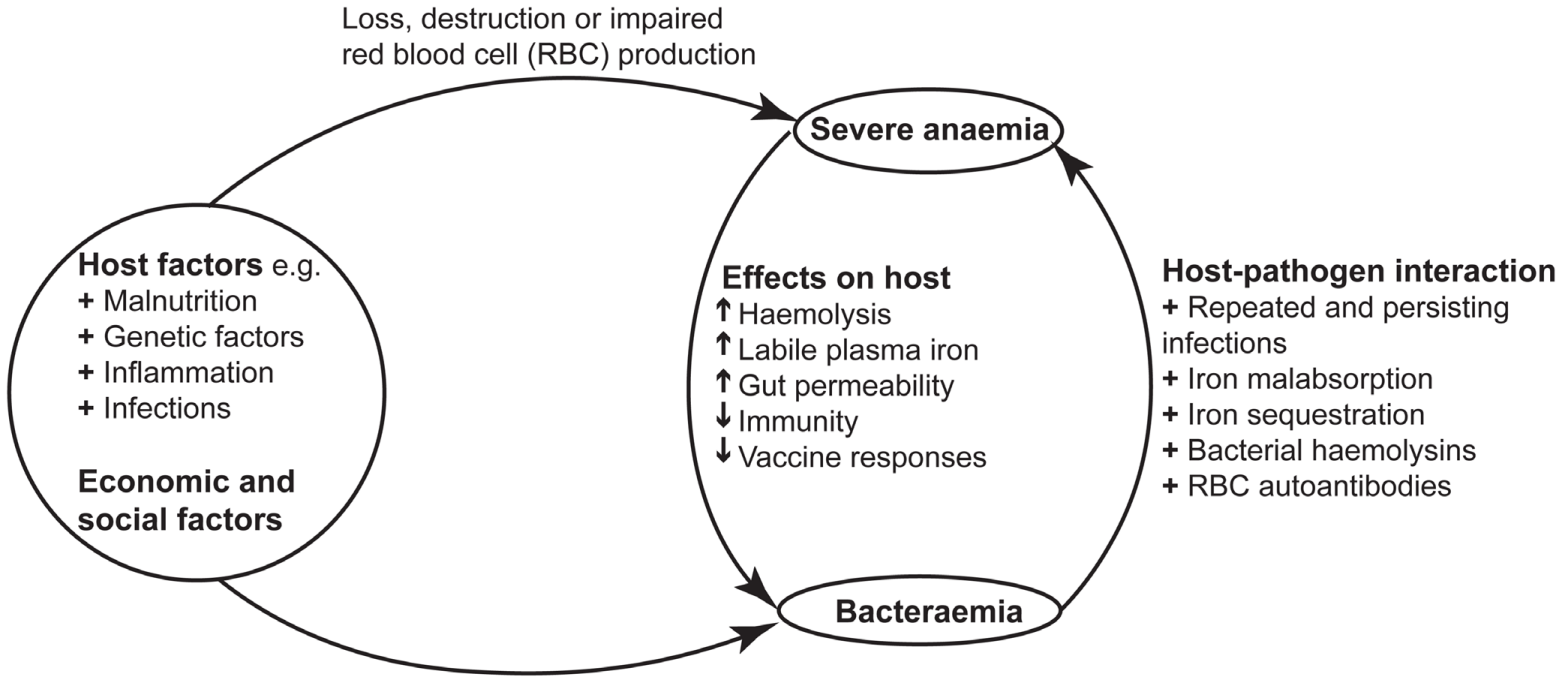
Association between severe anaemia and bacteraemia. In low-and-middle income countries, severe anaemia and bacteraemia often have similar and overlapping host and socio-economic aetiologies and associations.

Invasive bacterial infections remain important causes of paediatric in-hospital admission and mortality, and public health interventions remain imperfect. Invasive bacterial infections causing bacteraemia account for 6–15% of febrile hospital admissions with case fatality rates of 5–28% ^1– 5^ and a higher probability of post-discharge mortality ^6^. In sub-Saharan Africa, the commonest invasive bacterial pathogens are *Streptococcus pneumoniae, Staphylococcus aureus*, non-typhoidal *Salmonella* (NTS), *Haemophilus influenzae*, and *Escherichia coli* ^1– 3, 7^. In this region, susceptibility to invasive bacterial infections is sustained by a high prevalence of acquired comorbidities such as malnutrition, infections (malaria, and human immunodeficiency virus (HIV) infection), and sickle cell disease ^2, 8– 10^. A common feature among these comorbidities is the fact that they also cause severe anaemia ( Figure 1) ^11^, and accumulating evidence suggests a strong association between severe anaemia and increased risk of bacteraemia among African children ( Table 1).

**Table 1.** Association between severe anaemia and community-acquired bacteraemia in African children.

Country (Year published)	Region	Study subjects	Sampling dates	Sample size	Age range	Severe anaemia definition	No. with bacteraemia	Association and Effect size		Ref
								Severe anaemia	SMA	
Democratic Republic of Congo (2001)	Kivu	All admitted children	Jan 1989–Dec 1990	779	1–188 months	Hb <8 g/dL	124	P=0.001 ^1^	NR	4
Ghana (2015)	Agogo	Admissions with severe illness	May 2007–Mar 2011	1,915	<15 years	Hb <8 mg/dl	244	OR 2.5 (1.3, 4.5)	NR	26
Kenya (2011)	Siaya	Children with *P. falciparum* malaria	Mar 2004–Jan 2006	585	1–36 months	Hb <6 g/dL	59	NR	OR 1.56 (0.9, 3.0)	27
Malawi (2008)	Chikwawa & Blantyre	Primary diagnosis of severe anaemia	Jul 2002–Jul 2004	1,138	6–60 months	Hb <5 g/dL	68	OR 5.3 (2.6, 10.9)	NR	18
Mozambique (2009)	Manhica	All admissions	May 2001–Apr 2006	23,688	<15 years	PCV < 15% (<25% neonates)	1,550	OR 1.14 (0.9, 1.4)	NR	3
Nigeria (1993)	Benin City	Febrile (rectal temperature ≥38⁰C) preschool children without localizing signs	Oct 1988–Oct 1989	642	1 month–5 years	HCT ≤20%	67	χ ^2^ 0.24, P<0.7 (without malaria)	χ ^2^ 10.74, P<0.001	28
Uganda (2006)	Kampala	Severe malnutrition defined as symmetrical oedema or severe wasting	Sep–Nov 2003; Sep–Dec 2004	450	<60 months	Hb <5 g/dL	76	OR 2.3 (0.5, 10.2)	NR	10
Uganda (2020)	Jinja	Children admitted with severe anaemia	Jun 2016–Jan 2018	400	0–5 years	Hb <5 g/dL	3	P=0.47 ^1^	NR	29
Tanzania (2010)	Muheza	Admitted for febrile illness within the previous 48 hours	Jun 2006–May 2007	3,639	2 months–13 years	Hb <5 g/dL	349	OR 1.56 (1.1, 2.1)	NR	5

SMA denotes severe malarial anaemia; Hb, haemoglobin; PCV, packed cell volume; HCT, haematocrit; OR, odds ratio; aOR, adjusted odds ratio; and NR, not reported. ^1^Odds ratios not

Severe anaemia is a moderate-to-severe public health problem in many countries ^11^, and is associated with detrimental acute and long-term effects ^12– 14^. In sub-Saharan Africa, severe anaemia is prevalent among pre-school children ^15– 17^, and is present in 6–28% of febrile hospital admissions with case fatality rates of 4–10% ^18– 22^. Nearly one in five (18.8%) children with severe anaemia in developing countries die within 6 months of hospital discharge ^13, 22^. However, the biology of severe anaemia is complex. Several distinct mechanisms (including haemolysis, red blood cell production failure, and blood loss) lead to severe anaemia ^11^. Additionally, underlying aetiologies of severe anaemia, including infections (malaria, helminth infections, tuberculosis, and HIV infection), nutritional factors (acute/chronic malnutrition, and iron/folate/vitamin B _12_ deficiency) and haemoglobinopathies (such as sickle cell disease) often coexist in a single patient or population ( Figure 1). Although the World Health Organization has provided standard cut-offs for severe anaemia ^23^, definitions often vary across studies and populations ( Table 1). There are no internationally recommended cut-offs for diagnosing severe anaemia in children <6 months of age ^23^, and higher cut-offs (haemoglobin <9.0 g/dL) have been suggested for neonates ^17^.

## Severe anaemia disrupts iron homeostasis

Iron is an important nutrient required for the survival of nearly all aerobic organisms. The unique ability of iron to serve as an electron donor and acceptor renders it inimitable for various metabolic and physiological pathways, including cellular respiration, oxygen transport and DNA synthesis. In mammalian cells, iron is a co-factor for the assembly of functional iron-sulphur (Fe-S) cluster proteins, ribonucleotide reductases, and haem-binding proteins ^24^. The majority of the iron is intracellular, complexed within the porphyrin ring of haem or sequestered in ferritin, a heteropolymer capable of storing about 4,500 iron atoms. Despite its numerous benefits, excess “free” iron is toxic through its formation of reactive oxygen species (ROS) or hydroxyl radicals. As such, iron metabolism is highly regulated through control of its absorption, mobilization, storage, and recycling.

Iron is essential for haem and haemoglobin synthesis. In human adults, approximately 2.5×10 ^6^ red blood cells (RBCs) are produced per second ^25^. Accordingly, about 20–25 mg of iron is utilized for erythropoiesis daily. Most of the iron required for erythropoiesis is obtained from recycling of senescent and damaged RBCs by splenic red-pulp macrophages. In some conditions such as haemolysis, liver Kupfer cells and bone marrow erythroid island macrophages participate in iron recycling ^30, 31^. Dietary iron (about 1–2 mg) replaces iron lost through sweating, urinary and intestinal epithelial exfoliation, bleeding, or sloughing of epithelial cells. Dietary iron is absorbed in the form of haem (more bioavailable) or non-haem inorganic iron (mostly in the ferric (Fe ^3+^) form). Haem is absorbed more efficiently *via* receptor-mediated endocytosis ^32^ and is degraded by haem-oxygenase 1 (HO-1) into equimolar amounts of ferrous iron (Fe ^2+^), carbon monoxide, and biliverdin. Dietary inorganic Fe ^3+^ is first reduced to Fe ^2+^ by ferric reductase duodenal cytochrome B (DCYTB) ^33^. Fe ^2+^ is then transported across the apical membrane of enterocytes by the divalent metal transporter (DMT)-1 to form the intracellular labile iron pool ^34^. Some of the absorbed iron is stored in the enterocytes by ferritin while the rest is exported into the bloodstream *via* ferroportin (FPN), the main iron exporter, on the enterocyte basolateral membrane ^35^. This process is highly regulated by the hepatic iron-regulatory hormone hepcidin, which binds FPN and induces its internalization and endosomal degradation ^36^. Fe ^2+^ in the bloodstream is oxidised to Fe ^3+^ by hephaestin or caeruloplasmin. Fe ^3+^ binds to transferrin and is transported to the bone marrow, macrophages, hepatic stores or is used for other cellular processes ^37^. Some Fe ^3+^ binds to other ligands, such as albumin and citrate, to form the non-transferrin-bound iron (NTBI) pool (also known as labile plasma iron, or “free” iron) ^38^.

The process of iron absorption and recycling is highly adapted to maintain RBC production and optimal oxygen delivery to tissues. Severe anaemia, by acute or chronic blood loss or haemolysis, impairs this process. Rapid recovery from severe anaemia is thought to confer an evolutionary advantage ^39^, probably due to the critical role of haemoglobin in oxygen delivery to tissues. As such, an expanded erythropoietic drive up to eight-times the baseline is observed during chronic haemolysis or repeated haemorrhage ^40^. These high erythropoietic rates demand for increased iron supply. To achieve this, iron regulation by hepcidin is transcriptionally suppressed by erythroferrone (ERFE), a hormone produced by erythroblasts upon stimulation by erythropoietin (EPO) ^41, 42^. Other mediators such as platelet-derived growth factor (PDGF)-BB and soluble transferrin receptors could also suppress hepcidin production during severe anaemia ^43, 44^. Due to the evolutionary benefit of RBC restoration, hepcidin downregulation is observed to occur despite the presence of other underlying inflammatory or infectious mediators ^45– 47^.

In sub-Saharan Africa, severe anaemia is primarily caused by infectious agents (malaria, HIV infection, hookworms, and bacteraemia), sickle cell disease, and nutritional deficiencies (iron/folate/vitamin B12) ^11^. The nature and intensity of the disruption of iron homeostasis, therefore, varies depending on the underlying severe anaemia aetiology. For example, absolute iron deficiency, due to poor dietary iron uptake or inhibited iron absorption, is characterised by low levels of iron both in the plasma and hepatic stores. To restore RBCs, children with absolute iron deficiency require exogenous iron supplementation, but this may also predispose them to infections and risk of mortality ^48^. On the other hand, infectious agents are often associated with increased haemolysis (malaria, bacteraemia, and HIV infection) or haemorrhage (hookworm infections). During mild-to-moderate haemolysis, macrophages efficiently phagocytose damaged RBCs, break down haemoglobin and haem, and recover iron to sustain de novo erythropoiesis. Scavenger haemoproteins (such as haptoglobin and hemopexin) and receptors shuttle extracellular haemoglobin and haem to macrophages for clearance and detoxification ^49^. Pathological haemolysis, as expected with various severe anaemia aetiologies, results in sustained release of haemoglobin and labile haem which potentially surpass shuttle protein and macrophage clearance capacity. This results in increased plasma haemoglobin, labile haem, and iron, which are not only toxic, but can also accelerate the growth of invasive pathogens. Unliganded haem and haemoglobin can translocate across endothelial barriers into subendothelial and perivascular spaces and the lymph fluid ^50^. Furthermore, due to the need for iron in expanded erythropoiesis, iron regulation is impaired in severe anaemia. African children with severe anaemia and concomitant infections have markedly low hepcidin levels despite having elevated markers of inflammation ^45– 47^. This might also contribute to increased plasma iron levels, and potentially increase the pathogenesis of invasive bacterial infections.

## Iron is required for anti-bacterial immune responses

Iron is an important factor in the immune response to invasive infections. Iron deficiency protects against malaria ^51, 52^, while iron supplementation has been associated with increased risk of malaria, diarrhoea, and other infections ^48, 53^. However, these results have not been universally observed, and studies are often confounded or have marked biases ^54^. Little is known about the association of iron supplementation with bacteraemia incidence or mortality in endemic regions. Considerable literature has been published on how hepcidin sequesters iron away from invasive bacterial infections, especially in cell and mouse models ^55, 56^. This iron restriction, a main component of “nutritional immunity” ^57^, is effective against extracellular bacteria ^58– 60^, but could be detrimental for intracellular infections ^61^. Prolonged hepcidin upregulation also leads to mild-moderate anaemia of chronic disease ^62, 63^. In African children, anaemia may impair the development of immune responses from a young age ^64, 65^. Nonetheless, our knowledge of the effects of iron and anaemia (of varying severities) on cellular and non-cellular immune processes against bacterial infections remains incomplete.

### Innate immune responses

**Figure 2.**
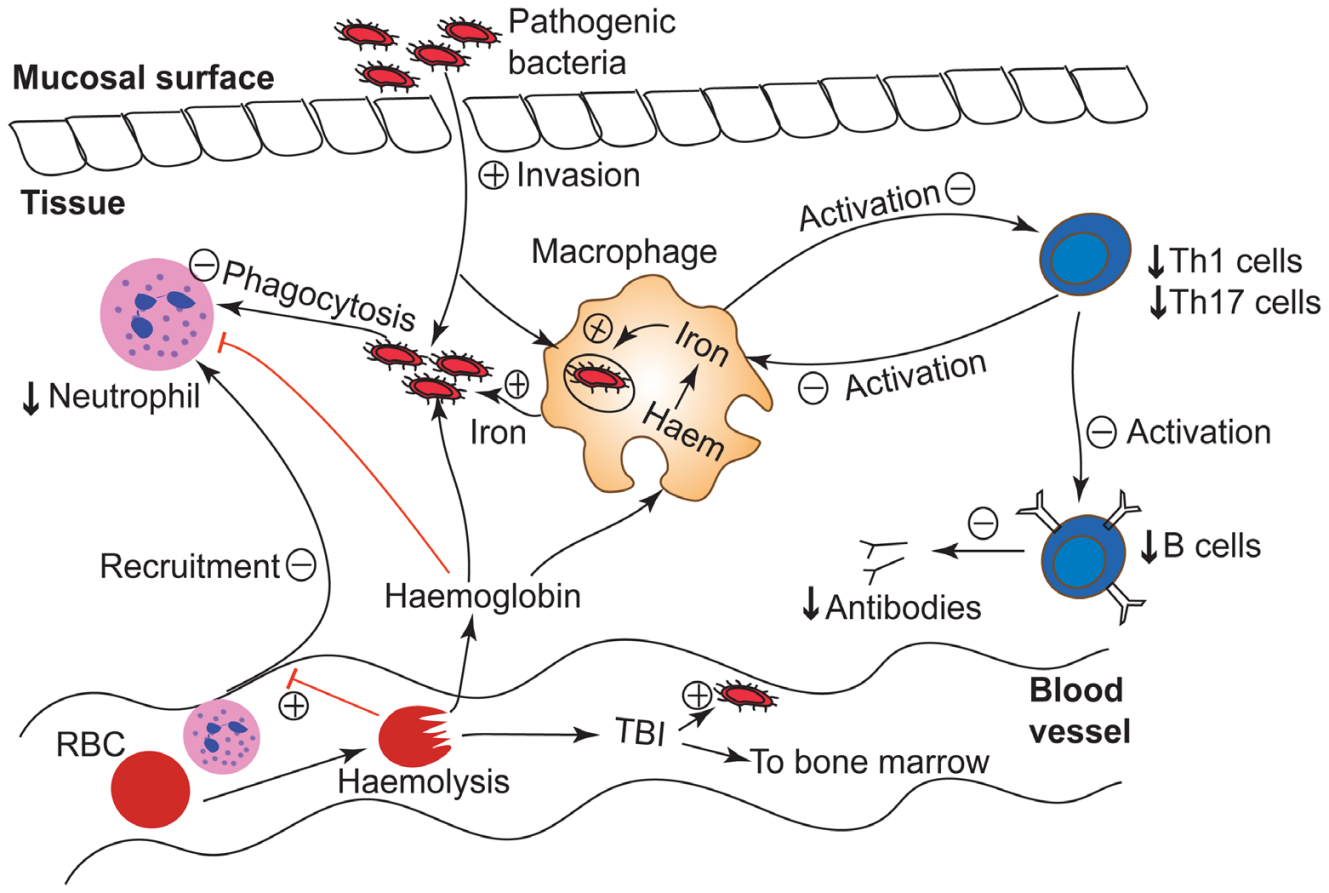
Potential mechanisms by which severe anaemia increases risk of bacteraemia. Severe anaemia increases the risk of invasive bacteria through increased gut permeability, impaired immune responses, and increased iron/haem availability to invading bacterial pathogens ( 65). Red arrows illustrate inhibitory pathways: + indicates increased effect and - indicates decreased effect. TBI denotes transferrin-bound iron; RBC, red blood cells, and Th, T-helper cells.

The skin and mucosal membranes provide a physical barrier against invasive bacterial infections. Breaches in these barriers (through burns, lacerations, abrasions, and wounds) provide access of bacteria, both commensal and pathogenic, to the bloodstream. Data suggest that severe anaemia promotes breaches in gut mucosal integrity. Severely anaemic neonatal mice had a persistent increase in intestinal permeability, which has been postulated to be due to abnormalities in epithelial adherens junctions ^66^ or decreased expression of the tight junction protein zonula occludens-1 ^67^. A similar increase in gut permeability has been reported in Kenyan children with severe malaria anaemia ^68^, although the precise mechanisms remain speculative. This gut barrier dysfunction, combined with possible microbiota dysbiosis during anaemia ^53^, feasibly underpin invasion of gut pathogens in children with severe anaemia ( Figure 2). The skin and mucosal membranes also play a role in systemic homeostasis through iron losses in the form of sweating, hair growth, and epithelial sloughing. A recent Mendelian randomization study of 48,972 European individuals found an association between higher iron status and risk of skin bacterial infections ^69^, possibly through increased iron supply to skin pathogens. Some antimicrobial peptides, such as lactoferrin and lipocalin-2 (also known as siderocalin or neutrophil gelatinase-associated lipocalin (NGAL)) sequester iron from bacterial pathogens on epithelial surfaces ^70, 71^. It is plausible that iron status also influences secretion of other epithelial antimicrobial molecules, such as cathelicidins, cryptdins, and β-defensins, but there is limited research in this subject.

Phagocytic leukocytes (macrophages and neutrophils) detect, ingest, and kill invasive bacterial pathogens. Pattern recognition receptors (PRRs) on phagocytic leukocytes detect bacterial pathogen-associated molecular patterns (PAMPs) such as lipopolysaccharide (LPS), unmethylated CpG DNA, and flagellin. The PRR–PAMP interaction induces a cascade of signalling events that mediate effector functions such as enhanced phagocytosis, macro-autophagy, proinflammatory cytokine and chemokine production, release of extracellular traps, antigen presentation, and generation of reactive oxygen species (ROS), reactive nitrogen intermediates and antimicrobial peptides ^72^. Iron status influences these PRR–PAMP interactions. For instance, ligation of the Toll-like receptor (TLR)-4, the LPS receptor, induces hepcidin production by macrophages and neutrophils ^73^. On the other hand, low intracellular iron levels in macrophages are associated with impaired activation of TLR-4 and reduced inflammatory cytokine expression ^74^.

Neutrophils, by virtue of their numbers and antimicrobial armamentarium, have the strongest antibacterial capacity. Consequently, the effects of iron status on neutrophil numbers and function will significantly influence bacterial control. Recent studies in mouse models found that neutrophil production and function are more iron demanding than production of other leukocytes ^75, 76^. Iron chelation and hepcidin-mediated iron deficiency substantially reduced neutrophil numbers, enhanced neutrophil extracellular traps (NETs), inhibited pharmacologically stimulated granulopoiesis, and suppressed antibacterial responses in mice ^76^. On the other hand, iron overload and increased labile haem availability, which are characteristic of haemolytic severe anaemia, also impair neutrophil antibacterial functions ^77, 78^. Mouse models of severe malarial anaemia have also reported haemolysis-associated reduction in neutrophil influx and lower production of proinflammatory cytokines ^78– 80^. This suggests a U-shaped relationship between iron status and neutrophil function, where both iron deficiency and iron excess are detrimental. In humans, iron deficiency is associated with neutropenia ^81^, neutrophil hyper-segmentation ^82, 83^, reduced neutrophil oxidative burst ^84^, and impaired NETs release ^85, 86^. Iron overload, characteristic of β-thalassemia major, is associated with impaired neutrophil phagocytosis and bactericidal activity ^87^, although this effect was not observed in iron overloaded hereditary hemochromatosis patients with low hepcidin levels ^88^. Previous studies found that monocytes from patients with HFE-associated haemochromatosis had low iron levels ^89, 90^, but little remains known on neutrophil iron status in these patients. Nonetheless, HFE-deficient mice demonstrated impaired neutrophil recruitment, suggesting a role for HFE in neutrophil responses ^91^.

Macrophages play a key role in nutritional immunity by controlling systemic and intracellular iron availability to invasive bacterial infections. Iron status itself influences macrophage function. Macrophages display a continuous spectrum of polarization, which influences their function. M1 macrophages (“classically-activated”) display the proinflammatory phenotype: they produce inflammatory cytokines (including interleukin (IL)-1β, IL-6, IL-12, IL-18, and tumour necrosis factor-alpha (TNF-α)), sequester iron into ferritin, and have potent antimicrobial capabilities. Increased intracellular iron promotes polarization to M1 phenotypes, and the molecular signature of proinflammatory M1 macrophages show increased activation of iron sequestration genes ^92^. Consistent with these observations, haemolysis and haem accumulation in hepatic macrophages trigger a proinflammatory phenotype in a mouse model of sickle cell disease ^93^. Although this phenotype is important for the control of extracellular bacteria, iron accumulation in macrophages is associated with the proliferation and persistence of intracellular pathogens such as *Salmonella enterica* spp., *Listeria* spp. and *Mycobacterium tuberculosis* ^94, 95^, while mechanisms of iron efflux from macrophages control these infections ^96^. M2 macrophages (“alternatively activated”) display the “healing” phenotype: they produce anti-inflammatory cytokines (IL-10 and transforming growth factor-beta (TGF-β)), actively release intracellular iron, and are involved in inflammation resolution and tissue repair. Iron release from M2 macrophages promotes cell proliferation, immune regulation, and matrix remodelling, consistent with their healing function. Acute iron deprivation of human macrophages is associated with reprogramming macrophages to the M2 phenotype in *in vitro* studies ^97^. 

Dendritic cells (DCs) are professional antigen presenting cells that create a bridge between the innate and adaptive immune responses. Once DCs phagocytose a pathogen, they become activated and transform to mature DCs, which migrate via blood to the spleen or via the lymphatic fluid to lymph nodes. Mature DCs upregulate expression of cell surface co-receptors (such as CD40, CD80 and CD86), which enhances their ability to activate CD4 ^+^ T cells, CD8 ^+^ T cells and B cells. Depending on their non-cognate costimulatory factors, DCs can also induce tolerogenic responses (unresponsiveness). Iron status can influence the numbers and function of DCs, and hence their ability to activate the adaptive immune responses. In *in vitro* studies, iron deprived monocytes differentiate into DCs with low expression of costimulatory molecules and are unable to activate T cells ^98^. In contrast, labile iron impairs the activation of hypoxia-inducible factor (HIF)-1α, a transcription factor critical in DC effector function. Sequestration of iron in ferritin counteracts this effect and promotes HIF-1α activation of DCs in response to LPS ^99^. In haemolytic anaemia, DCs also differentiate into macrophages to enhance erythrophagocytosis and iron recycling, reducing their numbers ^100^. This haem-induced differentiation of DCs to erythrophagocytes contributes to a secondary immunodeficiency in children with severe haemolytic anaemia. During bleeding or injury to the gut, DCs secrete hepcidin to sequester iron away from the microbiota in mouse models that helps with tissue healing ^101^. Nonetheless, there remains a general lack of research defining the influence of iron status, haem stress, and haemolysis on DCs in normal and disease states.

Non-cellular antimicrobial factors (such as complement, collectins, pentraxins, and ficolins) also form an integral part of the innate antibacterial immune responses. They bind to the bacterial PAMPs and trigger elimination of the invading organisms by direct lysis, agglutination, inhibition of growth, capsular swelling, opsonization, and activation of phagocytosis among other mechanisms ^102, 103^. Individuals with deficiencies in these factors, such as complement deficiencies, are more susceptible to bacterial infections. However, very little is known about the influence of iron status/anaemia on these antimicrobial factors. In healthy Asian adults, serum iron and ferritin levels were found to correlate with complement C3 and C4 levels ^104^, which conflicts with findings from an older study on 20 children which found no effect of iron deficiency on complement C3 levels ^105^. Since complement is an important bactericidal mediator, more definitive studies are needed to understand how its function is influenced by an individual’s anaemia and iron status.

### Adaptive immune responses

To successfully protect against invasive bacterial infections, the host must elicit efficient B and T cell responses. CD4 ^+^ T cells modulate responses by macrophages, neutrophils, CD8+ T cells and B cells. Cytotoxic CD8 ^+^ T cells directly kill bacteria-infected cells using molecules such as granzyme and perforin. B cells are key antibacterial cells by inducing antibody-dependent protection. Iron deficiency adversely affects the development and function of B and T cells. In some animal and cell models, iron deficiency was found to reduce the proportion of mature T cells, inhibit T cell proinflammatory cytokine release, and impair the proliferation and activation of T cells ^106– 108^. Loss of genes encoding iron regulatory proteins (IRP1 and IRP2) impairs iron uptake, proliferation, and effector functions of T cells ^106^. Additionally, activated T cells express more than one million copies of the transferrin receptor (TfR1) within 24 hours of antigen encounter ^106^, indicating an important role of transferrin-bound iron in T cell effector functions. Accordingly, *in vitro* blocking of TfR1 with antibodies inhibits polyclonal proliferation of human B and T lymphocytes ^107^. Absence of TfR1 or mutations in the TfR1 gene, *TFRC*, is associated with complete arrest of T cell differentiation and defects in lymphocyte activation ^106, 109^. Iron deficiency induced by chelation modulates the activity of DNA and histone demethylases, which impairs B cell proliferation ^110^.

In humans, genetic studies have found defective ex-vivo proliferation of B and T cells in patients with a hypomorphic mutation in the *TFRC* gene ^111^. *Ex vivo* addition of high concentrations of iron citrate rescued the proliferative defects. Iron deficiency is also associated with thymic atrophy, but findings are inconsistent ^112^. Two case–control studies, both with 40 children as cases, reported opposite findings on the effects of iron deficiency anaemia on B and T cell immunity ^113, 114^. One study found that cases (children with iron deficiency anaemia) had lower CD4 ^+^ counts and CD4/CD8 ratios, but similar immunoglobulin levels compared with the controls ^113^, while another study reported that cases had lower IgG levels but similar CD4/CD8 ratios compared with the controls ^114^. These contrasting findings can possibly be attributed to different definitions of iron deficiency, anaemia, and controls, and confounding by other infections and modifiable biological factors. Moreover, these findings underpin the complexity of understanding the effects of anaemia on immunity in heterogenous human populations (unlike mouse model studies) and indicate the need for more systematic approaches.

The cytokine milieu during a bacterial infection guides the functional polarization of CD4 ^+^ T cells, and subsequent antibody responses. A Th1 and Th17 skewed response (characterised by interferon-gamma (IFN-γ), IL-17, and IL-22 secretion) is required to control invasive bacterial pathogens. IFN-γ activates macrophages and induces polarization towards the proinflammatory M1 phenotype, while IL-17 and IL-22 recruit neutrophils to the site of bacterial infection. The M1 macrophages not only sequester iron away from invading pathogens, but also possess potent antimicrobial activity. Polymorphisms associated with increased IFN-γ production promote iron deficiency phenotypes ^115^. Interestingly, iron deficiency has been associated with reduced serum IFN-γ, IL-6, and TNF-α in animal and human studies ^108, 114, 116^, but these findings are not universally observed ^117^. Contrary to cell model studies ^104^, a cross-sectional study reported increased proportions of lymphocytes producing IFN-γ, TNF-alpha and IL-6 in participants with iron deficiency ^117^. These differences may be due to variations in study set-ups, severities of iron deficiency/ anaemia among the subjects, age, concomitant infections and/or underlying nutritional status. Many questions remain unanswered on the interaction of iron and adaptive immunity. For example, does iron supplementation in areas with high infectious disease burden improve immune responses? How do different severities of iron deficiency and anaemia affect B and T cell subset heterogeneity and long-term immune memory? Can these effects be reversed by iron supplementation?

## Iron status determines host–bacteria interactions

**Figure 3.**
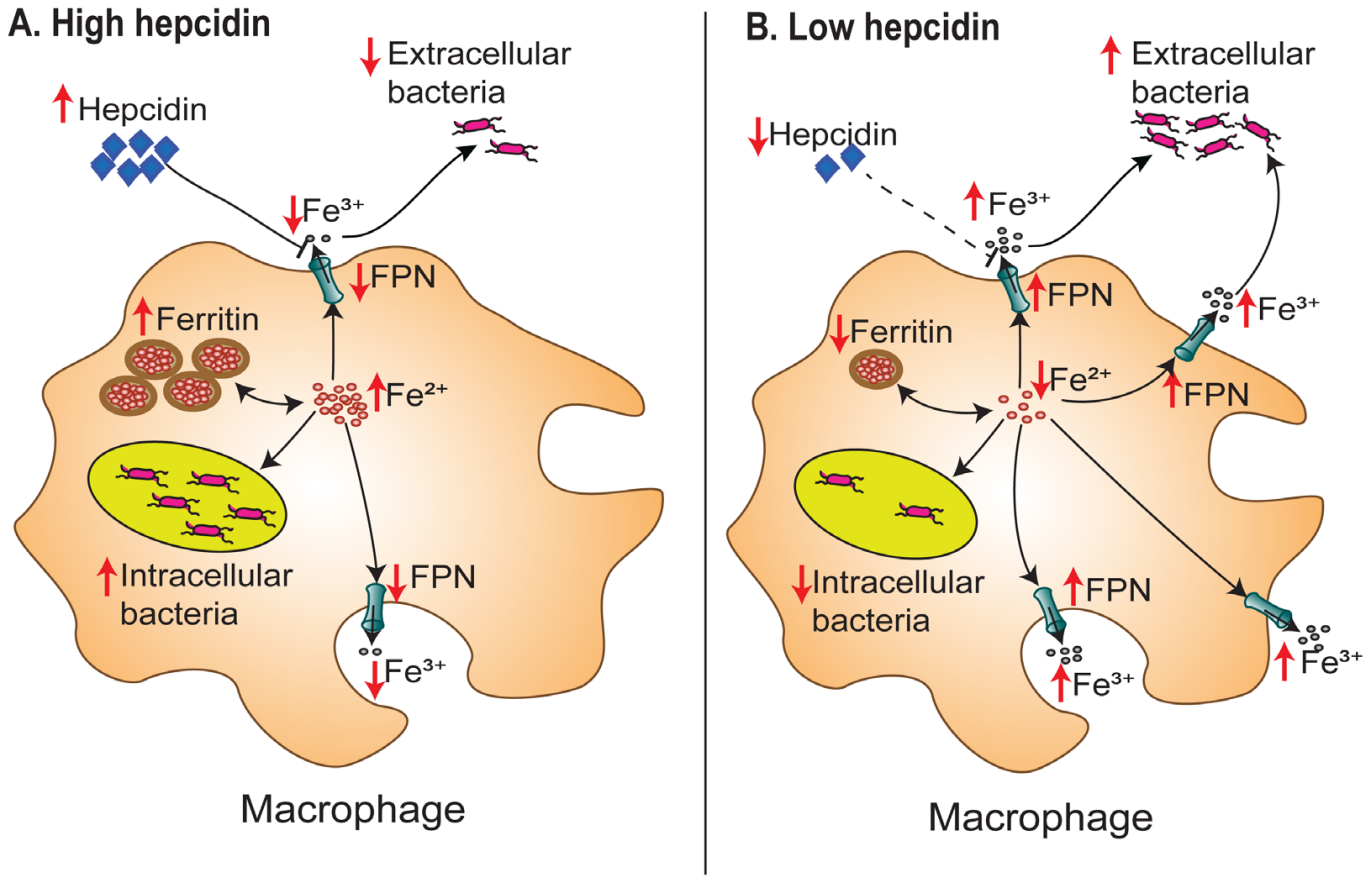
Association between iron status and bacteraemia. Hepcidin-mediated hypoferremia can protect against extracellular bacteria through iron sequestration, but this increases iron availability for intracellular bacteria. On the other hand, low hepcidin levels result in iron efflux from macrophages and starvation of intracellular bacteria but increases plasma iron availability for extracellular bacteria. FPN denotes ferroportin; Fe ^2+^, ferrous iron; and Fe ^3+^, ferric iron.

The competition for iron between the mammalian host and invading bacterial pathogens is important in determining the course and outcome of an infection ^55, 56, 118^. Not only is iron essential for the immune system, but iron is also required for bacterial growth and proliferation. Invading bacterial pathogens can access iron from various sources, including haem, haemoproteins, ferritin, transferrin-bound iron, non-transferrin-bound iron (NTBI), and lactoferrin ^119^. During a bacterial infection, the host traffics iron away from the site of infection, into intracellular iron stores (ferritin) to protect against extracellular pathogens or out of cells to protect against intracellular pathogens ^55^. The effectiveness of this iron sequestration is dependent on the bacterial niche and the bacterial iron acquisition strategies ( Figure 3). At the centre of this nutritional immunity is hepcidin, which blocks iron recycling by splenic macrophages and iron absorption by duodenal enterocytes and suppresses plasma iron concentrations ^120^. Hepcidin-mediated “hypoferraemia of infection” is effective against extracellular bacteria ^58– 60, 121^, but has been shown to promote the growth and proliferation of intracellular pathogens ^94, 122^. Other mechanisms that work independently or in collaboration with the hepcidin–FPN axis to redistribute iron away from invading pathogens include induction of lactoferrin, lipocalin-2, calprotectin, and the natural resistance-associated macrophage protein-1 (NRAMP1, also known as Slc11a1) ^70, 71, 123– 125^. Impaired iron regulation is associated with increased susceptibility to bacterial pathogens such as *Vibrio vulnificus* and *Escherichia coli* in iron overloaded patients with haemochromatosis and thalassemia ^126, 127^.

Following the start of a bacterial infection, proinflammatory cytokines such as IL-6, IL-1β and TNF-α induce the production of hepcidin ^128^. IFN-γ-mediated macrophage activation enhances intracellular bactericidal capabilities, including generation of ROS and reactive nitrogen intermediates, macro-autophagy, and nutrient deprivation ^72^. This includes iron efflux through increased FPN expression ^96^, a process that has been observed in mice infected with *Salmonella enterica* Typhimurium ^129^. Interestingly, elevated macrophage intracellular iron inhibits the activity of IFN-γ in a dose-dependent manner ^130, 131^. Intracellular bacteria are also starved of iron by siphoning out Fe ^2+^ from the bacteria containing vacuoles. This is thought to involve various iron transporters such as NRAMP1 and FPN ^124, 132^. However, the precise role of FPN in this process remains unclear, as recent studies have demonstrated that, based on its orientation, FPN transports iron into the *Salmonella*-containing vacuole ^133, 134^. This raises questions on the role of iron in the vacuoles – whether increased iron availability benefits intravacuolar bacteria or kills them through ferroptosis or the Fenton reaction. M2 macrophages express higher levels of FPN and have been reported to be the most preferred niche for *S. enterica* Typhimurium ^135^, suggesting an evolutionary advantage of infecting these cells. If FPN does indeed supply iron into the bacteria-containing vacuoles, then it is plausible that the iron benefits the bacteria and might explain the association of NTS with haemolytic severe anaemias in African children ^65^. The conflicting findings warrant further studies on intracellular iron homeostasis mechanisms during bacterial infections, especially in the context of iron deficiency and severe anaemia.

Invading bacterial pathogens have evolved numerous mechanisms to acquire iron from the mammalian host. The bacteria sense iron-limiting conditions and upregulate iron-acquisition and virulence genes ^56^. This is mediated by transcription factors, such as the iron-dependent ferric uptake regulator (Fur). In iron-limiting conditions, the Fur-mediated repression of iron transporters (such as the ATP-binding cassette (ABC) transporter) and siderophore genes is lifted, allowing enzymes for synthesis of iron acquisition proteins to be expressed ^136^. Siderophores, which are low-molecular-weight highly potent iron chelators, can extract Fe ^3+^ from transferrin and other human chaperone proteins ^137^. Bacteria can also acquire iron from haem, haemoproteins and siderophore-like haemophores ^56^. Gram positive bacteria, such as *Streptococcus pyogenes* and *S. aureus*, produce haemolysins and have membrane receptors that scavenge and bind myoglobin, haemoglobin, and haemoglobin-haptoglobin complexes ^138^. Haem from these haemoproteins is then transported through the envelope of bacteria into the cytoplasm, where it is degraded by haem oxygenase or reverse ferrochelatases to produce iron. Haem-responsive activators and receptors for haem, haemoglobin, and haemophores are also found on the outer membrane of some Gram-negative pathogens such as *H. influenzae*, *Yersinia enterocolitica*, *Yersinia pestis*, *Pseudomonas aeruginosa*, *Shigella* spp., *E. coli* O157:H7, *Bordetella* spp. and *Corynebacterium diphtheriae* ^139– 142^.

To safeguard against siderophores, the host relies upon lipocalin-2, which strongly binds enterobactin, the prototypical catecholate siderophore of many enteric bacteria, and sequesters the siderophore-iron complex ^71^. Mice lacking lipocalin-2 exhibit increased susceptibility to enterobactin-expressing bacteria ^143^. The host also sequesters labile plasma iron using lactoferrin, a high affinity Fe ^3+^ glycoprotein secreted by macrophages and neutrophils ^70^. Hemopexin clears labile haem from plasma, and expression of IL-22 after an infection elevates hemopexin levels ^144^. Bacterial defence against lipocalin-2 include use of “stealth” siderophores or production of competitive antagonists that bind lipocalin-2. Stealth siderophores, such as salmochelin ( *S. enterica* Typhimurium) and peterobactin ( *Bacillus anthracis*), are structurally modified making them resistant to lipocalin-2 ^145^. Pathogenic bacteria such as *Neisseria meningitidis and Neisseria gonorrhoeae* express lactoferrin receptors to bypass its iron restriction ^146^. Some bacteria also bind and extract iron from mammalian siderophore-like molecules including 2,5-dihydroxy-benzoic acid (DHBA) and dopamine ^147^.

The need for iron by bacterial pathogens presents an opportunity for vaccine development ^148^. In the iron “arms race”, bacteria have developed numerous conserved proteins that are expressed and exposed on surface membranes during an infection ^149^. Merck V710, a novel *S. aureus* vaccine targeting the highly conserved iron-scavenging protein IsdB, remains the most advanced vaccine targeting bacterial iron proteins ^150^. Although the vaccine was safe and demonstrated high immunogenicity in phase I studies ^151^, it was terminated during a phase II trial due to safety concerns and low efficacy ^150^. Participants receiving the Merck V710 vaccine had a higher mortality rate with post-operative *S. aureus* compared to those who received placebo ^152^. Despite this failure, the V710 vaccine proved that targeting bacterial iron proteins is a feasible vaccine approach against bacteraemia. Other proposed iron protein vaccine candidates include *Escherichia coli*’s siderophore receptor IroN and *N. meningitidis*’ enterobactin receptor FetA ^153, 154^.

## Severe anaemia increases the risk of invasive bacterial infections

An association between severe anaemia and invasive bacterial infections was reported long ago in a series of experiments by Kaye and colleagues in mouse models ^155– 157^. The researchers found that invasive *Salmonella* spp. was associated with severe anaemia induced by haemolytic processes (such as malaria infection and anti-RBC antibodies), but not non-haemolytic processes (phlebotomy) ^155– 157^. Subsequent mouse model studies have confirmed the association between haemolysis/iron status and *Salmonella* spp. bacteraemia ^77, 78, 158^. In humans, epidemiological studies have reported strong associations between severe anaemia and invasive bacterial infections ( Table 1), particularly with NTS bacteraemia ( Table 2). However, interpretation of these data is difficult as the studies were performed in different age-groups, used different definitions for severe anaemia, and were mostly underpowered. Some of the children might also have had HIV infection or undernutrition, which possibly confounded the associations observed in these studies. Additionally, severe anaemia aetiologies are heterogenous and complex, and data is not provided on other key predictors of bacteraemia such as socio-economic status, nutritional deficiencies, caregiver health status, and genetic factors ( Figure 1). Whereas iron deficiency is common in sub-Saharan Africa, prevalent in 70% of children in some areas ^159^, several studies have found no or negative associations between iron deficiency and severe anaemia ^18, 160, 167^. Consequently, most of the underlying aetiologies of severe anaemia in this region are haemolytic (except hookworm infections which cause severe anaemia through blood loss) ^11, 18^. It is, therefore, plausible that the observed association between severe anaemia and invasive bacterial infections is through haemolysis-associated mechanisms.

**Table 2.** Association between severe anaemia and community-acquired non-typhoidal *Salmonella* spp. bacteraemia in African children.

Country (Year published)	Region	Study subjects	Sampling dates	Sample size	Age range	Severe anaemia definition	No. with NTS	Association and Effect size		Ref
								Severe anaemia	SMA	
Democratic Republic of Congo (2016)	Oriental	Body temperature ≥38°C or ≤35.5°C; suspicion of severe localized infections; or suspicion of sepsis, typhoid fever, and severe malaria	May 2009–May 2014	3,467	All ages	Hb <5 g/dL	113	OR 5.07 (0.6, 41.4)	NR	175
Kenya (2022)	Kilifi	All paediatric admissions	Aug 1998–Oct 2019	75,034	≤60 months	Hb <5 g/dL	400	aOR 3.15 (2.4, 4.2)	aOR 2.17 (1.4, 3.3)	46
Kenya (2006)	Kilifi	All admissions with 1 *Salmonella* spp. bacteraemia	Aug 1998–Jul 2002	16,570	<13 years	Hb ≤5 g/dL	166	OR 3.0 (2.1, 4.3)	NR	176
Malawi (2007)	Blantyre	Admissions with severe malaria and had blood culture results	1996–2005	1,388	≥6 months	PCV <10%	14	NR	aOR 4.5 (1.8, 11.5)	177
Malawi (2002)	Blantyre	All children 1) with a final diagnosis of severe malaria; 2) who received a blood transfusion	Feb 1996–Jun 1999	701	3–148 months	PCV ≤15%	18	NR	aOR 3.86 (1.3, 1 1.5)	178
Malawi (2000)	Blantyre	All sick children with a positive blood isolate of NTS species	Feb 1996–Apr 1998	299	3 days–14 years	PCV ≤15%	299	RR 7.2 (3.4, 15.3)	NR	179
Malawi (2000)	Blantyre	All children with clinically suspected bacteraemia	Sep 1996–Aug 1997	10,508	1 day–14 years	PCV <16%	140	RR 3.55 (1.68, 7.49)	NR	180
Mozambique (2015)	Manhica	All children <2 years, and children 2 to <15 years with axillary temperature ≥39.0°C or signs of severe illness	Jan 2001–Dec 2014	51,878	<15 years	PCV <15%	670	5.0 (3.5, 7.2)	NR	181
Mozambique (2009)	Manhica	All admissions with *Salmonella* spp. bacteraemia	May 2001–Apr 2006	23,688	<15 years	PCV <15% (<25% neonates)	401	aOR 3.48 (2.0, 5.95)	NR	182
Tanzania (2014)	Moshi and Muheza	A history of fever in the past 48 hours, an axillary temperature ≥37.5°C, or a rectal temperature of ≥38.0°C	Jun 2006–May 2007; Sep 2007–Aug 2008	4,106	2 months–13 years	Hb <5 g/dL	163	aOR 2.19 (1.5–3.2)	NR	183
Tanzania (2010)	Muheza	Fever of ≥3 days prior to admission or fever of <3 days but with at least 1 severity criteria	Mar 2008–Feb 2009	1,502	2 months–14 years	Hb <5 g/dL	45	P<0.0001 ^2^	NR	184
Multi-country ^1^ (2016)	Multi-centre	Febrile patients with a tympanic temperature ≥38.0°C or axillary temperature ≥37.5°C	Mar 2010–Jan 2014	13,431	All ages (except Ghana, ≤15 years were enrolled)	Hb <7 g/dL (Hb <8 g/dL for ≥5 years)	73	aOR 14.62 (5.6, 38.4)	NR	185

SMA denotes severe malarial anaemia; NTS, non-typhoidal Salmonella; Hb, haemoglobin; PCV, packed cell volume; OR, odds ratio; aOR, adjusted odds ratio; NR, not reported; RR, risk ratio. ^1^Countries in the study include Burkina Faso, Ethiopia, Ghana, Guinea-Bissau, Kenya, Madagascar, Senegal, South Africa, Sudan, and Tanzania. ^2^ Odds ratios not reported.

Haemolytic anaemias induce sustained release of labile haem and NTBI. Haem impairs the recruitment and function of phagocytic leukocytes, including their ability to kill ingested bacterial through ROS ^162, 163^. In extreme haemolysis, haem is toxic to tissues, and may cause immune paralysis hence impeding resistance to invasive bacterial infections ^163^. HO-1, the haem-catabolizing enzyme, induces tolerogenic effects of the immune system, and impairs resistance to NTS and other pathogens ^164– 166^. Haem breakdown products, biliverdin and carbon monoxide, scavenge antibacterial radical molecules and contribute to anti-inflammatory responses ^167^. Increased NTBI levels are associated with increased susceptibility to siderophilic extracellular bacteria in mouse models ^59, 60^, and we have previously hypothesized that increased NTBI and labile haem increase NTS risk in Kenyan children with severe malaria ^46^. We demonstrated that young children with severe malaria anaemia, accounting for more than 50% of admissions with severe anaemia in the study area, had markedly suppressed hepcidin levels compared to other forms of malaria, suggesting that the association between severe anaemia and bacterial infections may be through iron-mediated pathways ^46^. Very low hepcidin levels could impair the host’s ability to withdraw iron from invading bacterial pathogens as observed in mouse models ^58– 60^. It is also plausible that the acute need of iron for erythropoiesis promotes polarization of macrophages to the iron releasing M2 phenotype, which has poor antibacterial activity ^72^.

**Figure 4.**
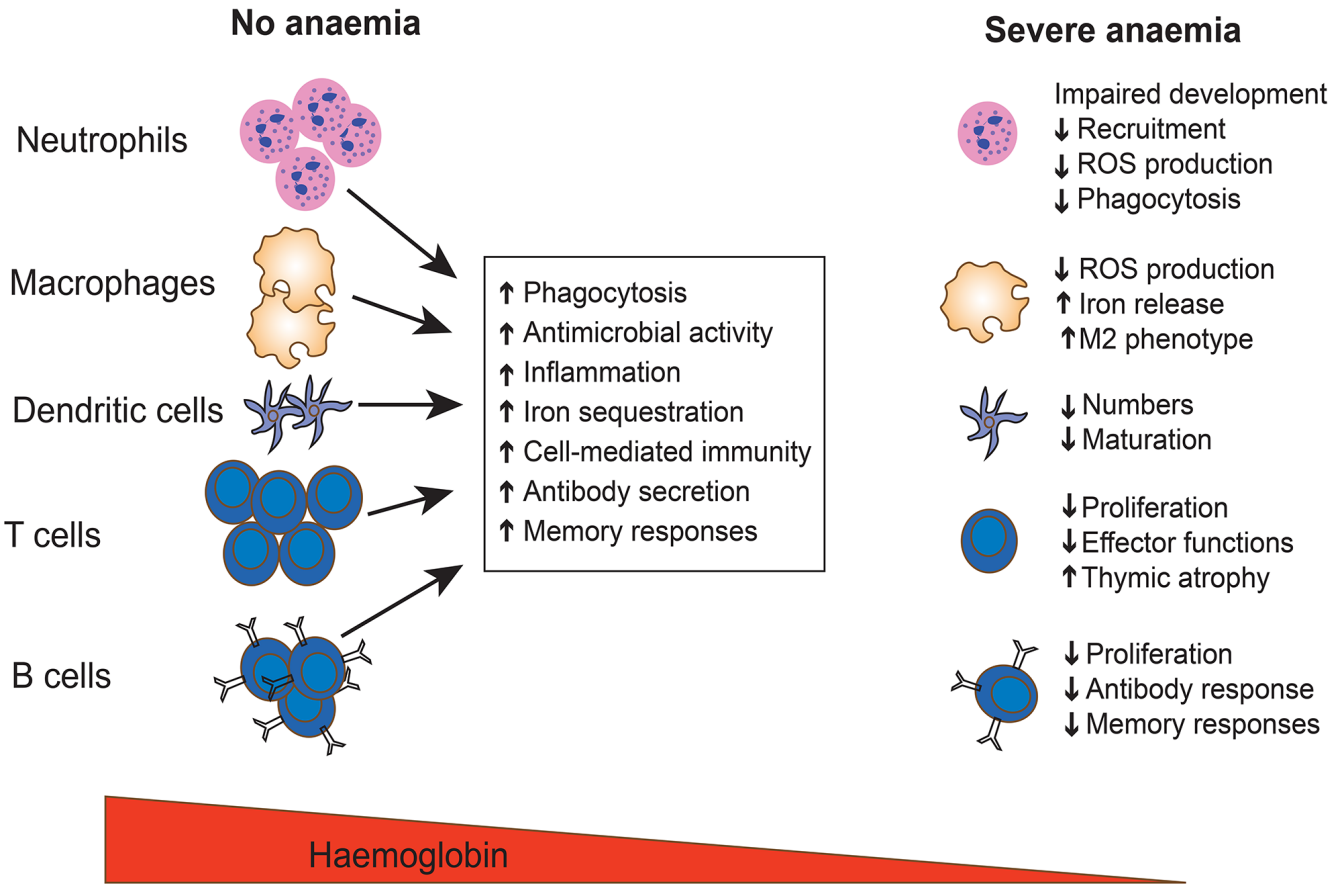
Effects of severe anaemia on the immune system. Severe anaemia impairs the development, recruitment, and function of key immune cells required in the elimination of invasive bacteria. ROS denotes reactive oxygen species.

Severe anaemia also has direct and profound adverse effects on cellular immune responses to bacteraemia ( Figure 4). Early studies found that children with low haemoglobin levels had lower proportions of T cells and humoral bactericidal capabilities ^168, 169^. Evidence from literature suggests that these disruptions of immune function increase along a spectrum of anaemia severities and is dependent on the underlying severe anaemia aetiology ^65^. For example, malarial anaemia reduces neutrophil and macrophage count, impairs oxidative burst capacity, reduces neutrophil bactericidal activity, and promotes iron accumulation in macrophages ^77, 158, 170, 171^. Severe malarial anaemia is also associated with blunted production of IL-12 ^172^, a cytokine critical for control of NTS bacteraemia ^173^. Although not epidemiologically associated with severe anaemia in sub-Saharan Africa ^18, 160, 161^, severe iron deficiency is associated with neutrophil and macrophage dysfunction, cytokine dysregulation, impaired antigen-specific antibody production, reduced mature B-cell populations, thymic atrophy, and poor T cell responses ^76, 106, 110, 116^. Consequently, disruption of iron status impairs immune responses to bacterial infections, and as previously discussed, there is possibly a U-shaped relationship between iron status and immune function. In mouse models, iron supplementation of anaemic mice was associated with increased *S. enterica* Typhimurium colony-forming units in the spleen ^174^. Both iron deficiency and iron overload, which are characteristic of different aetiologies of severe anaemia, negatively affect the development, recruitment, and function of various immune cells as discussed in this review. The availability of haem and its products during severe haemolysis exacerbates this immune impairment and may result in immune paralysis in extreme cases. Mechanistic studies of the association between severe anaemia and invasive infections should factor in specific anaemia aetiologies to help in the development of targeted interventions. Complexities arise in situations of overlapping aetiologies, such as haemolysis in children with severe iron deficiency. These circumstances are more common in malaria-endemic and resource limited settings than is appreciated, and little is understood regarding how the body regulates iron or the immune outcomes of bacterial infections in these patients.

## Severe anaemia and iron deficiency impair immune responses to bacterial vaccines

Vaccines are extremely effective public health interventions. Licensed bacterial vaccines in routine immunisation schedules have drastically reduced the incidence and mortality from invasive pathogens such as *C. diphtheriae*, *Bordetella pertussis*, *Clostridium tetani*, *H. influenzae* type b, *S. pneumoniae*, and *N. meningitidis*. There is considerable progress in vaccine development for other bacterial pathogens using emerging technologies such as structural vaccinology, novel adjuvants, reverse vaccinology, and rationally designed bacterial outer membrane vesicles ^186, 187^. While vaccines have made an unprecedented impact on human health, not everyone benefits from vaccine protection equally. Poor vaccine efficacy is characteristic of low-income populations where severe anaemia and iron deficiency are highly prevalent ^188^, and it is plausible that severe anaemia and/or iron deficiency impair vaccine responses. Most licensed bacterial vaccines confer protection by eliciting long-lasting protective antibody responses. Higher vaccine efficacy depends on synergy of B and T cell responses, as well as innate immune responses. However, iron deficiency and severe anaemia have widespread adverse effects on major immune cells required for efficient vaccine response ( Figure 4).

Data on the effect of severe anaemia and/or iron deficiency on bacterial vaccine responses remain scarce, and the little available is mostly of poor quality ^112, 189^. We did not find records of any clinical trial that has investigated the effect of iron supplementation on bacterial vaccine efficacy. Observational studies on typhoid, diphtheria, and tetanus vaccines found no association between iron deficiency anaemia and vaccine-induced antibody titres ^105, 168, 190, 191^, although iron deficient children had a lower T cell response ^191^. Nonetheless, the studies included very few subjects and were likely underpowered ^189^. In a recent study on a cohort of Kenyan children, iron deficiency and anaemia at the time of routine childhood vaccination were strong predictors of poor responses to diphtheria, pertussis, and pneumococcal vaccines, while moderate and severe anaemia were the strongest risk factors for anti-diphtheria sero-negativity at 18 months after vaccination ^192^. This is consistent with a study from Ecuadorian infants that found low anti-diphtheria antibodies following DTP vaccinations among anaemic children ^193^. Further studies are needed to confirm and quantify these associations between anaemia/iron deficiency and poor vaccine efficacy in other areas with a high burden of anaemia/iron deficiency.

In the context of severe anaemia and/or iron deficiency, there are some noteworthy concerns about vaccine efficacy that have not been addressed. First, it is not known whether severe anaemia or iron deficiency reduce antibody affinity or mediate wrong specificity of antibodies during germinal centre maturation, and if this is dependent on the severity of anaemia or iron deficiency. Second, while data show that iron is required for T and B cell proliferation activity, it is not known what quantity of serum iron is critical, and whether increased haemolysis or haem stress affects these processes. Germinal centre reactions are fundamental for the generation of high-quality and long-lasting B cell responses, and more studies are needed to understand how anaemia or iron status influences germinal centre responses in humans. More data is also needed to determine the effects of anaemia/iron status on vaccine responses including antibody avidity, T cell polarization, cytokine production, timing of immune responses, and their effects on the microbiome. Retrospective analysis of data from vaccine trials in areas with high anaemia and iron deficiency burden would provide invaluable insights, but this is contingent on such studies not having excluded participants with anaemia or iron deficiency. Standardised measurements and definitions of iron deficiency, accounting for inflammation and malaria ^159^, are also required in these regions, as incorrect prevalence estimates may undermine the analysis and interpretation of data.

## Conclusion

Severe anaemia and invasive bacterial infections are strongly associated among children living in sub-Saharan Africa, and the association may be due to disruption of iron regulation during severe anaemia. The aetiology and severity of anaemia are important determinants of the course and outcomes of invasive bacterial infections. However, our understanding of the interaction between anaemia, iron, and immune responses remains incomplete. Most of the existing evidence on anaemia, iron and immunity stems from *in vitro* and mouse model studies, and the findings are mostly conflicting due to differences in experimental set-ups. Extrapolating results from these controlled laboratory set-ups to heterologous human populations in anaemia endemic areas remains challenging. There is also limited information on the effects of anaemia and iron status on vaccine efficacy, even though areas and populations with high anaemia and iron deficiency are also the ones with poor vaccine efficacy to currently available vaccines. As a result, there is an urgent need for more reliable and systematically collected data from endemic areas to understand how anaemia and iron status influence immune responses to infections and vaccination, and whether this differs by anaemia aetiology and severity.

## Data Availability

No data are associated with this article.
