# Supplementary material for: Severe anaemia, iron deficiency, and susceptibility to invasive bacterial infections
Source: Wellcome Open Res. 2023 Feb 2;8:48. [Version 1] doi: 10.12688/wellcomeopenres.18829.1 (PMC10439361; doi:10.12688/wellcomeopenres.18829.1)
Supplement: Peer review history

# Peer review: Reviewer response for version 1

**DOI:** 10.21956/wellcomeopenres.20875.r61498

**Published:** 2023-08-18

**Authors:** Gaetano Cairo

**Affiliations:** 1 Department of Biomedical Sciences for Health, Universita degli Studi di Milano, Milan, Lombardy, Italy

The field of iron metabolism has shown great development over the last years, and in particular the link with inflammatory diseases is emerging. Therefore, it is timely to review the complex pathogenetic mechanisms linking iron with anemia and bacterial infections, which is an increasingly important issue both in the bio-iron field and human health, particularly in sub-Saharan Africa and in children. The authors submitted a clearly written manuscript that appears accessible also to a non-specialized audience. Figures are well conceived and help visualize important information for scientists outside of the area. Overall, the review is clear and well balanced, providing an excellent coverage of the topic. I have no major concerns, but some suggestions for revision are provided below.

 It is well-known that hypoferremia represents an immune “nutritional” mechanism against siderophilic bacterial pathogens that, if prolonged, may lead to anemia.  Inflammatory mediators induce the iron-regulatory hormone hepcidin, which blocks ferroportin-dependent iron absorption and recycling. Iron uptake into the mononuclear phagocyte system and storage in the form of ferritin are stimulated. This response deprives extracellular infectious agents from circulating iron sources, but inevitably results in a functional iron deficiency and directs iron fluxes away from the erythron, which is the major iron consumer. Therefore, when inflammatory stimuli persist, functional iron deficiency in the erythron leads to anemia.

 In this Review, the Authors point out that anemia is not only a consequence of chronic inflammation. In fact, they provide ample evidence that the reverse situation may occur: severe anemia impairs the function of the immune cells required for the elimination of invasive bacteria. Indeed, several key immune cells, such as different types of macrophages, neutrophils and various classes of T cells require iron for optimal growth and performance in a number of inflammatory settings. Both the innate and adaptive immune response are involved.

 The molecular regulation of this competition for iron between pathogens and immune cells is mediated by hepcidin, which is induced by inflammation. On the other hand, when anemia occurs, erythropoietin-dependent increase in the expression of erythroferrone is expected to downregulate hepcidin. The Authors could comment further on this issue. Is the balance between these contrasting factors depending on the intensity of the different stimuli ? Does it depend on the various pathophysiological settings (different types of infection) ? Why inflammatory induction of hepcidin is prevailing in most circumstances 

**Minor point**

 Paragraph *Severe anaemia disrupts iron homeostasis *(page 3): the sentence “excess free iron is toxic through its formation of reactive oxygen species (ROS) or hydroxyl radicals” is not entirely correct. Actually, ferrous iron catalyses the transformation of relatively harmless products of cell metabolism like superoxide and hydrogen peroxide in the highly reactive hydroxyl radical.

Is the review written in accessible language?

Yes

Are all factual statements correct and adequately supported by citations?

Yes

Are the conclusions drawn appropriate in the context of the current research literature?

Yes

Is the topic of the review discussed comprehensively in the context of the current literature?

Yes

Reviewer Expertise:

Iron metabolism-erythropoiesis-inflammation

I confirm that I have read this submission and believe that I have an appropriate level of expertise to confirm that it is of an acceptable scientific standard.

# Peer review: Reviewer response for version 1

**DOI:** 10.21956/wellcomeopenres.20875.r55343

**Published:** 2023-03-24

**Authors:** Jason P. Mooney

**Affiliations:** 1 Institute of Immunology and Infection Research, The University of Edinburgh, Edinburgh, Scotland, UK

The authors provide a comprehensive review for an important, yet complex disease. Still, the text is clear and easy to read – both for non-experts and new researchers.  In this review, Abuga et al. bring together the fields knowledge of severe anemia, iron deficiency, and invasive bacterial infections. While the list of comorbidities which are associated with invasive bacterial infection is long and well documented ( *e.g. malnutrition, malaria, HIV, SCD*), the authors instead focus on one aspect, anaemia.

 They approach this review by discussing the impact of anemia on iron metabolism, followed by a cell by cell breakdown of both innate and adaptive immune cells. Each paragraph/section could be expanded into its own review. Yet, the authors provide a good summary here. As the authors state, the current data the mechanism(s) of these interactions is incomplete. 

 Rather than delving into the details between studies, the authors bring together data for the wider picture. Further, the complexities of the aetiologies for the population at risk would make this comparison difficult, in such a broad review as this. Still, I personally enjoyed the information provided in the tables. For example, Table 1 highlights discrepancies in the data – where the definition of severe anemia can vary and the association with malaria not reported.

 I think the review is well structured and timely, and drives the message that a more comprehensive understanding is needed, particularly in future prospective clinical studies.

Is the review written in accessible language?

Yes

Are all factual statements correct and adequately supported by citations?

Yes

Are the conclusions drawn appropriate in the context of the current research literature?

Yes

Is the topic of the review discussed comprehensively in the context of the current literature?

Yes

Reviewer Expertise:

Immunology; malaria; invasive salmonellosis

I confirm that I have read this submission and believe that I have an appropriate level of expertise to confirm that it is of an acceptable scientific standard.
